# Identifying 14-3-3 interactome binding sites with deep learning

**DOI:** 10.1039/d5dd00132c

**Published:** 2025-08-08

**Authors:** Laura van Weesep, Rıza Özçelik, Marloes Pennings, Emanuele Criscuolo, Christian Ottmann, Luc Brunsveld, Francesca Grisoni

**Affiliations:** a Institute for Complex Molecular Systems (ICMS), Eindhoven University of Technology Eindhoven The Netherlands l.brunsveld@tue.nl f.grisoni@tue.nl; b Eindhoven AI Systems Institute (EAISI), Eindhoven University of Technology Eindhoven The Netherlands; c Centre for Living Technologies, Alliance TU/e, WUR, UU, UMC Utrecht Utrecht The Netherlands; d Laboratory of Chemical Biology, Department of Biomedical Engineering, Eindhoven University of Technology Eindhoven The Netherlands

## Abstract

Protein–protein interactions are at the heart of biological processes. Understanding how proteins interact is key for deciphering their roles in health and disease, and for therapeutic interventions. However, identifying protein interaction sites, especially for intrinsically disordered proteins, is challenging. Here, we developed a deep learning framework to predict potential protein binding sites to 14-3-3 – a ‘central hub’ protein holding a key role in cellular signaling networks. After systematically testing multiple deep learning approaches to predict sequence binding to 14-3-3, we developed an ensemble model that achieved a 75% balanced accuracy on external sequences. Our approach was applied prospectively to identify putative binding sites across medically relevant proteins (ranging from highly structured to intrinsically disordered) for a total of approximately 300 sequences. The top eight predicted peptide sequences were experimentally validated in the wet-lab, and binding to 14-3-3 was confirmed for five out of eight sequences (*K*_d_ ranging from 1.6 ± 0.1 μM to 70 ± 5 μM). The relevance of our results was further confirmed by X-ray crystallography and molecular dynamics simulations. These sequences represent potential new binding sites within the 14-3-3 interactome (*e.g.*, relating to Alzheimer's disease as the binding to tau is not the new part), and provide opportunities to investigate their functional relevance. Our results highlight the ability of deep learning to capture intricate patterns underlying protein–protein interactions, even for challenging cases like intrinsically disordered proteins. To further the understanding and targeting of 14-3-3/protein interactions, our model was provided as a freely accessible web resource at the following URL: https://14-3-3-bindsite.streamlit.app/.

## Abstract 2

Protein–protein interactions drive many biological processes. A deep learning model was developed to predict putative 14-3-3 binding sites. Experimental validation through binding assays and crystallographic studies confirmed novel interactions.

## Graphical abstract

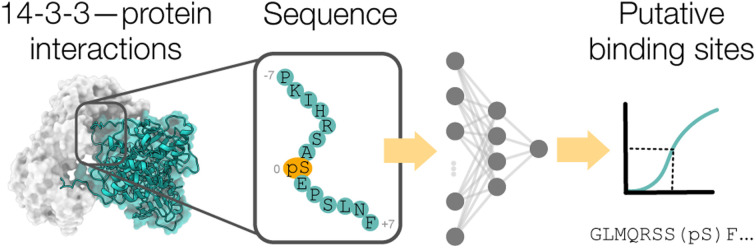

## Introduction

Protein–protein interactions (PPIs) are fundamental to all biological processes, from maintaining cellular homeostasis^1^ to driving disease mechanisms.^2^ Among the numerous protein families facilitating PPIs, the family of 14-3-3 proteins stands out due to their ubiquity and high conservation across isoforms.^3,4^ These ‘central hub’^5^ proteins hold a key role in cellular signaling networks, as they are known to interact with over 1200 protein clients,^6,7^ and are involved in pathways related to metabolism, apoptosis, cell signaling and tumor development. Protein interaction with 14-3-3 can yield a multitude of effects,^8^*e.g.*, the structural stabilization of the client protein,^9,10^ the masking of functional sequences,^11,12^ or bringing two proteins together.^13,14^ Owed to these reasons, elucidating the 14-3-3 interactome (protein clients and/or their binding sites) has a key relevance to gain insights into cellular regulation and mechanisms of disease, as well as to provide new avenues for therapeutic intervention.

While it is important to identify 14-3-3 binding partners and their binding sites, it is a daunting task. Proteins can interact with each other in a wide variety of ways, and the exact protein interaction sites and corresponding interaction effects are often unknown.^15^ Combinatorial exploration in the wet-lab is both costly and time intensive.^16–18^ Owed to these reasons, deep learning^19^ – a subfield of artificial intelligence based on neural networks – has gained significant traction to predict PPIs.^20–23^ Deep learning, thanks to its ability to extract complex and non-linear information from large and high-dimensional data,^19^ bears promise to accelerate the identification of unknown binding sites involved in PPIs. To date, however, deep learning approaches have found only limited experimental validation in exploring protein interactomes,^20,24^ and only a few approaches have focused on protein interactions with 14-3-3,^25,26^ or phosphorylated proteins in general. Furthermore, 14-3-3 proteins interact with multiple and diverse intrinsically disordered phosphorylated targets,^27^ which challenges the usage of established deep learning approaches that rely on protein structure to perform a prediction.^23^

Stemming from these observations, this work aims to aid in binding site identification to explore the 14-3-3 protein interactome, by leveraging deep learning on peptide sequences. Our approach was designed to predict putative sites of proteins binding to 14-3-3. After training our model on publicly available data, and benchmarking it in comparison with existing models,^26^ we validated it prospectively in the wet-lab. *Via* a combination of model interpretation, crystal structure determination, and molecular dynamics, we show the potential of the proposed approach to prioritize putative interaction sites of proteins with 14-3-3.

## Results and discussion

### Predicting binding to 14-3-3 with deep learning

#### Study setup

**Fig. 1:**
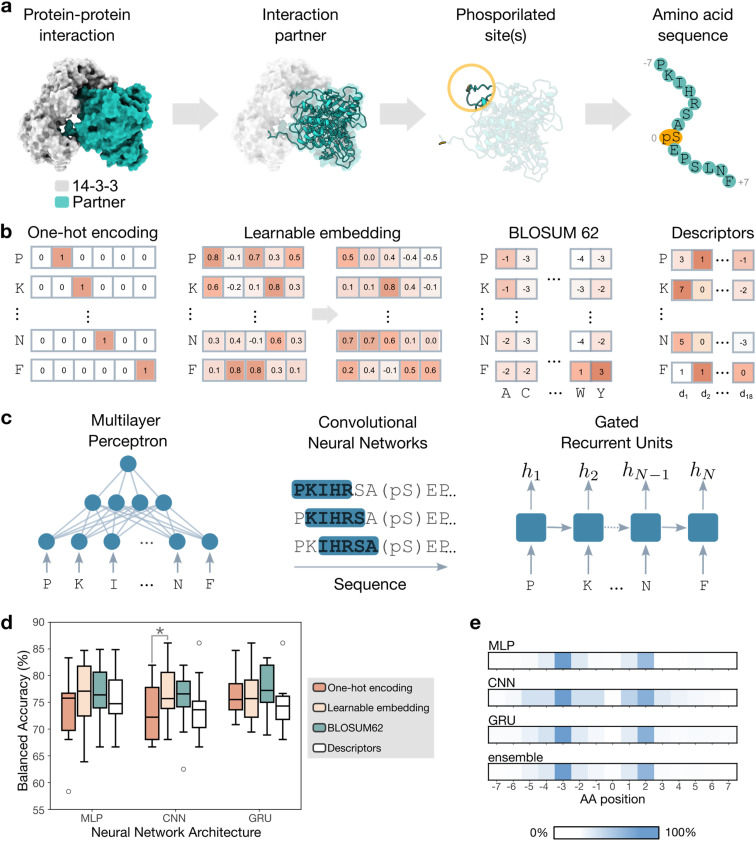
Predicting peptide sequence binding to 14-3-3 with deep learning. (a) The information on tested 14-3-3 protein interactions (Table 1) was converted into a ‘machine readable’ format. Seven amino acids (AAs) before and after the phosphorylated site were used to form a sequence for deep learning, which is labeled according to its binding to 14-3-3 (yes/no). (b) Representations of AA sequences for model training. One-hot encoding represents each AA with a unique binary vector. Learnable embedding starts with a random numerical vector per AA and updates the vectors during training. BLOSUM62 uses substitution scores derived from evolutionary conservation. Descriptors are pre-defined features capturing the physico-chemical properties of each AA. (c) Neural network architectures. Multilayer perceptron (MLP) consists of fully connected layers. Convolutional neural networks (CNNs) slide windows over the input sequences, and gated recurrent units (GRU) iterate over the input AAs in a stepwise manner. (d) Balanced accuracy per architecture-representation combination (computed on 10 test sets obtained *via* repeated splitting). Statistically significant differences are marked with “*” (paired Wilcoxon test, *α* = 0.05). (e) Interpretation of the best models *via* input perturbation. By randomly shuffling all AAs in any given position, we computed the relative change in the model predictions. Color indicates the relevance of the perturbation in each position, normalized by maximum achieved change, ranging from 0% (white: no impact) to 100% (blue: maximum impact).

Predicting PPI sites with machine learning is a challenging endeavor, especially when dealing with intrinsically disordered proteins, like the typical 14-3-3 binding partners.^27^ In these cases, structure-based approaches inevitably fail.^28^ To this end, the prediction task was cast into modeling the 14-3-3 interaction with the individual binding sites of known clients using their amino acid (AA) sequences (Fig. 1a). We used an existing dataset^26^ (Table 1), and represented each binding site as a peptide sequence comprising the seven AAs preceding and seven AAs following the phosphorylated site (for a total of 15 AA per binding site, Fig. 1a). Moreover, an additional set of 92 phosphopeptides measured in-house for their binding to 14-3-3 was used for model validation (Table 1).

**Table 1:** Datasets used in this study, along with the number of AA sequences contained, and their labels (binders, non-binders). The publicly available dataset was used for model training and selection, and the in-house set for external validation

Dataset	No.	Binders	Non-binders
Training/validation set^26^	715	360 (50%)	355^a^ (50%)
In-house set	92	58 (63%)	34 (37%)

a 93 experimentally determined (26%), and 262 (74%) likely non-binders.

#### Model training and benchmarking

The publicly available data was used for model training, and it was split ten times into training (67.5%), validation (22.5%), and test sets (10%). We represented the AA sequences numerically using four approaches (Fig. 1b):

• One-hot encoding, where each AA in the peptide is represented as a binary vector indicating its type. This representation captures the information in the sequence without introducing prior assumptions or additional knowledge about the AAs.^29^

• Learnable embeddings, where AAs are assigned different, randomly initialized vectors.^30^ These embeddings are updated during training to help capture contextual and relational information about the AAs in the sequence.

• BLOcks SUbstitution Matrix (BLOSUM 62),^31^ where AAs are encoded with substitution matrix scores, which reflect evolutionary conservation and property similarities between AAs. This representation incorporates biochemically relevant information about AA substitutions. Phosphorylated AAs were indicated *via* a dedicated binary flag in the corresponding position (see Materials and methods).

• Physico-chemical descriptors, where each AA in the peptide is represented by 18 pre-computed numerical features^32^ (SI Table S1). For each peptide, the computed AA features were concatenated and used for the prediction.

Each representation was combined with the following deep learning architectures (Fig. 1c):

• Multilayer perceptron (MLP),^33^ where complex peptide features are progressively extracted through multiple layers of fully-connected neurons,^34^ without explicitly considering positional information.

• Convolutional neural network (CNN),^35^ in which windows (‘kernels’) slide over an input sequence, and learn to weight input elements at each window. CNNs capture local patterns in sequences, which are combined to predict the global properties of a sequence (*e.g.*, binding).

• Recurrent neural network with gated recurring units (GRU),^36^ which iterates over the input sequence and encodes information from the N- to the C-terminus, compresses the information into a ‘hidden state’, which is then used to provide a prediction.

For each representation-architecture combination, we performed hyperparameter tuning and evaluated the model on the 10 test sets (obtained *via* stratified splitting). The best model for each combination was evaluated on the test sets using balanced accuracy (BA), which captures the global model performance (Materials and methods, eqn (4)). In general, no statistically significant difference between model architectures was observed (Wilcoxon signed rank test, *α* = 0.05). Moreover, the chosen sequence representations were the main drivers of performance, with different trends based on the chosen architecture (Fig. 1d). For each architecture, we chose the representation leading to the highest balanced accuracy (average over 10 test-set splits), resulting in: (a) MLP with learnable embedding (BA = 77 ± 4%); (b) CNN with BLOSUM 62 encoding (BA = 73 ± 5%); and (c) GRU with BLOSUM 62 encoding (BA = 78 ± 6%). Moreover, an ensemble model was obtained by averaging the prediction of each model. While this model did not improve the overall balanced accuracy (BA = 77 ± 5%), it increased the capacity to correctly recognize binding sequences, as shown by an increased recall (SI Table S2).

The models were then retrained with all available data. Their performance was benchmarked in comparison with 14-3-3-Pred.^26^ 14-3-3-Pred also combines three machine learning approaches (MLP, support vector machine [SVM], and position-specific scoring matrix [PSSM]) into an ensemble model. Both 14-3-3-Pred and our models were validated on the in-house set (92 peptides, Table 1), as it comprises peptides external to all considered models and exhibiting diverse recurring AA motifs (SI Fig. S1) – hence allowing us to assess the potential for prospective validation. In addition to balanced accuracy, we calculated the capacity of the models to minimize false positives (precision) and to correctly recognize binding and non-binding sequences (recall and specificity, see Methods, eqn (1)–(3)). The models developed in this work systematically outperformed 14-3-3-Pred in global performance (balanced accuracy), and in most cases in terms of identification of true positives (recall, Table 2). Moreover, they consistently ranked second-best in the ability to minimize false positives (precision and specificity, Table 2). Finally, the ensemble approach balanced the strengths and weaknesses of each individual deep learning model.

**Table 2:** Model benchmarking on an external test set. Our model was compared with an existing one (14-3-3-Pred) on a set of 92 external peptides, across various classification metrics: balanced accuracy (BA), precision (Pr), recall (Rc), and specificity (Sp) (Methods, eqn (1)–(4)). For each classification metric, the best and second-best performance are highlighted in boldface and with italics, respectively

Model	Approach	BA (%)	Pr (%)	Rc (%)	Sp (%)
This work	MLP (learnable)	71	81	*84*	59
	CNN (BLOSUM 62)	71	*82*	75	*67*
	GRU (BLOSUM 62)	*73*	*82*	*84*	63
	Ensemble	**75**	*82*	**91**	59
14-3-3-Pred^26^	MLP	60	74	71	48
	SVM	61	**89**	29	**93**
	PSSM	60	74	71	48
	Ensemble	65	*82*	64	*67*

#### Model interpretation

To shed light onto the binding patterns learned by the models, we conducted a virtual mutation study. We randomly shuffled (15 times) AAs occurring in each position, except for the phosphorylated AA, of the training peptides and used each model to predict the binding probability of the ‘virtually mutated’ sequences (Fig. 1e). The AAs comprised between −5 and +3 positions contributed the most to the predictions across models, in alignment with previous findings.^26^ Moreover, the AAs in the −3 and +2 positions yielded the largest average change in predictions when perturbed. This is in line with structural biology observations, as the occurrence of arginine and proline at these positions is the most common binding motif for the interaction with 14-3-3.^16^ Finally, each modeling approach has a ‘prediction hallmark’, with a main focus on the AAs in the −5 and +3 position. Additional differences exist, albeit they are not particularly marked for CNN and GRU (both based on BLOSUM62 representation, Fig. 1e). This suggests that, although the individual models are trained on the same data, they might capture slightly different sequence-binding relationships. This might contribute to the increased performance of the ensemble model for most metrics (Table 2).

### Prospective model application

#### Experimental validation of binding sites to 14-3-3

We applied our model prospectively to identify putative, previously unidentified, binding sites with 14-3-3. As a case study, we selected seven medically relevant proteins: forkhead box O3 (FOXO3),^37^ Tau,^38^ Myc,^39^ Bcl-2-associated agonist of cell death (BAD),^40^ Notch-4,^41^ Cystic fibrosis transmembrane conductance regulator (CFTR),^42^ and p53.^43^ These proteins contribute to a wide array of cellular processes^37,40,44^ (*e.g.*, metabolism, cell survival and death) and are involved in diseases like cancer^45,46^ (*e.g.*, BAD, p53 and Notch-4), Alzheimer's (Tau) and cystic fibrosis (CFTR).^47^ The structures of these proteins range from ordered (CFTR and Notch-4: experimental/predicted disorder ratio^48,49^ between 0% to 26%) to partially and highly disordered (p53, Myc, FOXO3, BAD, Tau; experimental/predicted disorder ratio^48,49^ ranging from 38% to 95%, SI Table S3). Hence, they constitute an interesting and diverse test case for the 14-3-3 interactome.

For the selected proteins, their AA sequence was obtained from UniProt.^50^ All serine and threonine residues were localized and a sequence window of 15 AAs was obtained (−7 and +7 around such AAs), leading to a total of 830 sequences. These sequences were further analyzed with PhosphositePlus^51^ to verify whether they are phosphorylated *in vivo*. Only sequences labeled as phosphorylated (either according to literature^41^ or to PhosphositePlus) were retained, resulting in a library of 296 peptides. These sequences were ranked by the ensemble model for their predicted binding to 14-3-3. Importantly, our model identified known binding sites for all proteins (13 in total, across Tau, BAD, FOXO3, Notch-4, CFTR, Myc and p53; SI Table S4), further corroborating the predictive ability and applicability of our approach.

**Fig. 2:**
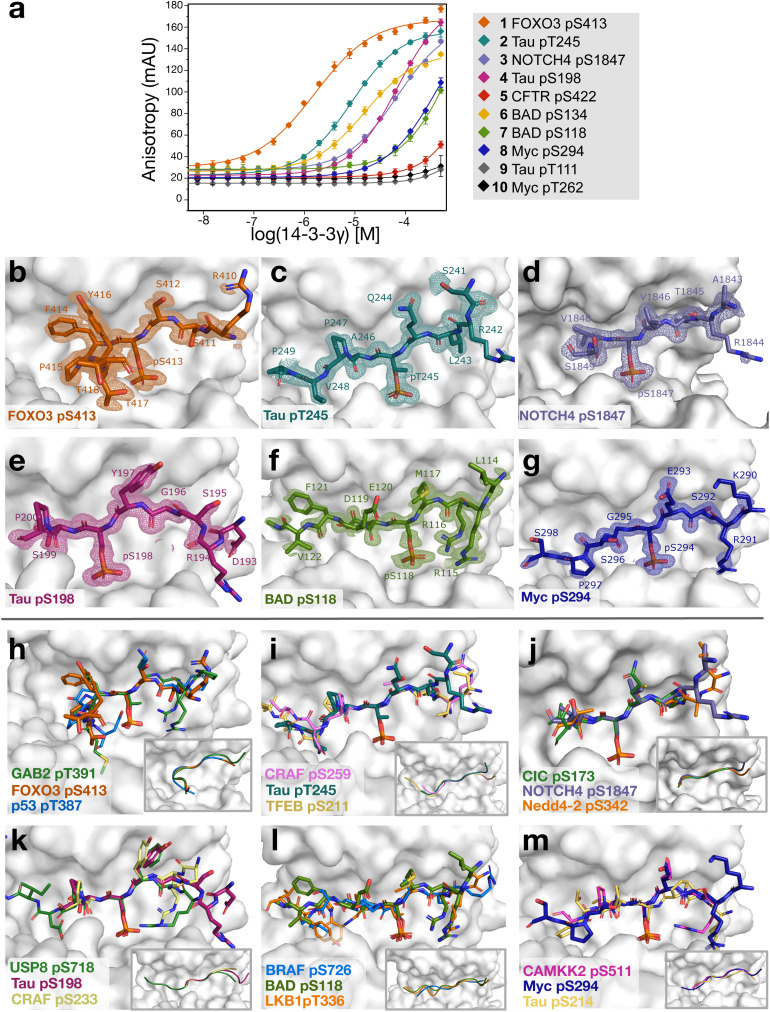
Experimental characterization of the selected peptides. (a) Characterization *via* a fluorescence anisotropy assay. Binding curves are reported on a logarithmic scale for each sequence, labeled as protein and phosphosite (pS = phosphoserine, pT = phosphothreonine), across three independent repeats. (b–g) X-ray crystallography on selected peptide binders in comparison with known binders. Crystal structures of the predicted peptide sequences (colored sticks) in complex with 14-3-3sigma (white surface). Final 2*F*_o_ − *F*_c_ electron density contoured at 1.0*σ*. (b) FOXO3 pS413 (orange), (c) Tau pT245 (cyan), (d) NOTCH pS1847 (purple), (e) Tau pS198 (pink), (f) BAD pS118 (green), (g) Myc pS294 (blue). (h–m) Crystallographic overlay of predicted peptide sequences (h) FOXO3 pS413 (orange), (i) Tau pT245 (cyan), (j) NOTCH pS1847 (purple), (k) Tau pS198 (pink), (l) BAD pS118 (green), (m) Myc pS294 (blue) with two known 14-3-3 binding peptides (colored sticks) in the 14-3-3 pocket (white surface). Each figure includes a representation of the peptide backbones.

From the model predictions, we filtered out the known binders, and selected eight top-scoring sequences, first ranked based on the majority vote of the ensemble model, and then by average prediction score across the three models (1–8, Table 3). Moreover, two bottom-scoring sequences were picked as negative controls (9–10, Table 3). These peptide sequences were obtained with a N-terminal fluorescent label to measure their binding affinity to 14-3-3γ *via* fluorescence anisotropy (FA) assays (Fig. 2a). Three out of eight ‘positive’ peptides (37%) showed strong, low-micromolar binding affinities (as measured *via* their dissociation constant [*K*_d_], Table 3), equal to *K*_d_ = 1.6 ± 0.1 μM (1, FOXO3 pS413), *K*_d_ = 8.6 ± 0.8 μM (2, Tau pT245), and *K*_d_ = 15.9 ± 1.9 μM (6, BAD pS134). The remaining positive sequences showed binding, albeit weaker (*K*_d_ ranging from 70 μM to larger than 100 μM), except for the CFTR-pS422 peptide, which showed no binding in the FA assay (Fig. 2a). As expected, the negative controls 9 and 10 did not bind, confirming the correctness of the model-based ranking.

**Table 3:** Peptide selection and validation. Eight putative binding sites and two negative controls were selected for experimental validation, using the model predictions. Peptides 1–8 were selected by maximizing the predicted binding score, while peptides 9–10 were selected as negative controls (predicted to be non-binding with high confidence). Predicted outcome (binding and non binding, using a threshold above 0.5 in the predicted binding score) is also reported. The protein, phosphosite, AA sequence (pS = phosphoserine, pT = phosphothreonine) and model predictions are reported, along with the experimentally determined constant of dissociation (*K*_d_ [mean ± SD, *n* = 3]). Binding curves are reported in SI Fig. S2

ID	Protein	Phosphosite	AA sequence	Model		*K*_d_ (μM)
				Predicted outcome	Predicted binding scores	
1	FOXO 3	413	GLMQRSS(pS)FPYTTKG	Binding	0.98 ± 0.02	1.6 ± 0.1
2	Tau	245	SAKSRLQ(pT)APVPMPD	Binding	0.94 ± 0.05	8.6 ± 0.8
3	Notch 4	1847	FPRARTV(pS)VSVPPHG	Binding	0.87 ± 0.08	70 ± 1
4	Tau	198	SGDRSGY(pS)SPGSPGT	Binding	0.85 ± 0.06	71 ± 11
5	CFTR	422	NNNNRKT(pS)NGDDSLF	Binding	0.69 ± 0.14	—
6	BAD	134	KGLPRPK(pS)AGTATQM	Binding	0.68 ± 0.14	15.9 ± 1.9
7	BAD	118	GRELRRM(pS)DEFVDSF	Binding	0.65 ± 0.11	>100
8	Myc	294	APGKRSE(pS)GSPSAGG	Binding	0.61 ± 0.04	>100
9	Tau	111	EEAGIGD(pT)PSLEDEA	No binding	0.000 ± 0.0005	—
10	Myc	262	LHEETPP(pT)TSSDSEE	No binding	0.001 ± 0.0009	—

Interestingly, peptide 2 (Tau-pT245) showed higher binding affinities than the known 14-3-3 interaction sites:^52,53^ Tau-pS214 (*K*_d_ = 16.4 ± 0.9 μM) and Tau-pS324 (*K*_d_ > 100 μM; SI Fig. S3). This is especially interesting considering that Tau-pT245 is reported to be only phosphorylated in a normal, non-Alzheimer brain.^54^ The FOXO pS413 peptide showed similar affinity to the known FOXO pS253 site.^55^ The p253 site is located near the nuclear locator site, whilst pS413 is located close to the nuclear exclusion site,^56^ suggesting a potential dual role by 14-3-3.

The experimental *K*_d_ values correlate well with the predicted binding scores (*r* = 0.74). Discrepancies exist for peptides with moderate predictions (scores ranging from 0.69 to 0.61), such as 5 (CFTR pS422) and 6 (BAD pS134). In this case, no evident relationship between AA similarity to training sequences and predicted binding scores exists. While this success ratio is in line with literature on machine learning for peptide discovery,^57–59^ it underscores intrinsic model limitations, *e.g.*, due to coverage of sequence-binding relationships, experimental error, and the presence of likely non-binders in the training set (Table 1). Moreover, binding data used for training come from diverse experimental assays, potentially contributing to differences with the reported FA measurements.

Moreover, when comparing our predictions with those of 14-3-3-Pred^26^ and with 14-3-3 Site Finder, we observe moderate to no correlations between the predictions on the selected sequences (ranging from 0.17 to 0.61). Finally, the ranking obtained by our model correlates well with the observed *K*_d_ predictions (0.74, SI Table S5). Additionally, when comparing the peptides to the most common 14-3-3 binding motifs (RSXpSXP,^16^ RXY/FXpSXP,^16^ pS/pT (X1–2)-COOH,^60^ with X being any AA), seven out of eight sequences (except for sequence 1) would not have been found. These results corroborate the predictivity of our approach and its relevance to rationalize sequence binding to 14-3-3 beyond known common motifs.

#### X-ray crystallography

The binding of the selected sequences was further confirmed and molecularly probed by X-ray crystallography through co-crystallization of 14-3-3 with peptides 1–8 (Fig. 2). Crystal structures were obtained for nearly all peptides that demonstrated binding in the FA assay, except for BAD pS134. These experiments validated the interaction of the newly discovered phosphorylated peptides to 14-3-3, as evident from the electron density maps which reveal the conformation of the peptides within the 14-3-3 binding pocket (Fig. 2b–g). Structural overlays with previously characterized 14-3-3/peptide complexes show that the binding modes of these predicted peptides are comparable to known interactions, indicating that these sequences are likely physiologically relevant rather than artificial (Fig. 2h–m).

The FOXO3 pS413 peptide exhibits an ‘open’ binding mode, bending outward from the 14-3-3 pocket due to a proline residue at the +2 position (Fig. 2b). A similar binding conformation was observed for the GAB2 pT391 peptide,^61^ which aligns perfectly at its +2 proline with FOXO3, and for p53 pT387,^62^ which bends out of the pocket due to glycine and proline residues at the +2 and +3 positions, respectively (Fig. 2h). The high affinity of FOXO3 pS413 can be attributed to key interactions at the protein–peptide interface, including hydrogen bonds between FOXO3 residues S411 and S412 and 14-3-3 residues D225, N226 and W230 (Fig. S5a). Additionally, FOXO3 F414 interacts with the hydrophobic roof of the 14-3-3 pocket composed of L218, I219, and L222. A network of water-mediated hydrogen bonds is formed between the FOXO3 backbone and K49, K122 and N175 of 14-3-3. The phosphorylated residue of FOXO3 (pS413) is also involved in this hydrogen bond network, thereby stabilizing the bent conformation of the peptide (SI Fig. S4a). The high-affinity binding of FOXO3 pS413 was further corroborated by molecular dynamics simulations on the peptide (and the sequence extended by 40 AAs within the full-FOXO3 protein, see Materials and methods), showing consistently low root mean squared fluctuation (RMSF) values (SI Fig. S5).

Despite also containing a +2 proline, the Tau pT245 peptide adopts a distinct binding mode, extending further into the 14-3-3 pocket (Fig. 2c). The ‘extended’ binding mode is similar to peptides such as CRAF pS259 ^63^ and TFEB pS211,^64^ all of which fold back into the pocket after a minor turn induced by the +2 proline (Fig. 2i). Conformational variations at the N-terminal side of the phospho-residue were observed, though the electron density in this region was not well-defined. Notably, all newly identified peptide sequences contained a positively charged arginine at the −3 or −4 position, consistent with many known 14-3-3 client peptides. The binding mode of Tau pT245 was also confirmed by molecular dynamics simulations on the tested peptide sequence and its extended version (with 40 additional AAs, see Materials and methods and SI Fig. S5b and c). In this context, Tau pT245 exhibited limited fluctuations in its interactions with 14-3-3 over time, as assessed by RMSF analysis (SI Fig. S5a).

For NOTCH4 pS1847, electron density was only observed up to the +2 serine, suggesting that the remaining residues are disordered (Fig. 2d). Similar C-terminal disorder has been reported in crystal structures of the 14-3-3 clients CIC pS173 ^65^ and Nedd4-2 pS342 ^66^ (Fig. 2j). In addition, only the +1 and +2 residues were resolved in the Tau pS198 crystal structure (Fig. 2e). The −1 tyrosine residue of Tau pS198 was observed in previously reported structures of USP8 pS718 ^67^ and CRAF pS233,^68^ where it fits into a pocket at the top of the 14-3-3 binding groove (Fig. 2k).

Although BAD pS118 and Myc pS294 exhibited the weakest binding affinities among the tested peptides, their crystal structures displayed more ordered C-terminal regions compared to Tau pS198 and NOTCH4 pS1847 (Fig. 2f and g). The +1 aspartate residue of BAD pS118 is interacting with K122 of 14-3-3, followed by a +3 phenylalanine that shields the negatively charged aspartate (SI Fig. S5e) – an arrangement that appears unique among known 14-3-3 binding proteins, as far as we know. Therefore, the structural overlay for the BAD pS118 crystal structure shows more variation in the C-terminal side of the peptide (Fig. 2i). Nevertheless, some similarities were revealed in the overlay with BRAF pS726 ^69^ and LKB1 pT336,^70^ where BRAF's +1 glutamate aligns with BAD's +2 glutamate, and LBK1's +3 proline and +5 leucine occupy the same pocket as BAD's +3 phenylalanine. Moreover, molecular dynamics simulations on the extended version of BAD pS118 (by 20 residues on the N- and -C terminus of the original BAD sequence) show improved stabilization, compared to the shorter peptide, especially visible from the −4 leucine to the −2 arginine residues. These analyses further support pS118 as a putative binding site of BAD to 14-3-3.

Finally, the Myc pS294 peptide forms a slight turn within the 14-3-3 pocket due to its +1 glycine, similar to CAMKK2 pS511 ^71^ and Tau pS214,^72^ where this turn is induced by a +2 proline (Fig. 2g and m). This leads to a highly comparable binding mode among the 14-3-3 client peptides. In conclusion, the predicted binding sites of clinically relevant 14-3-3 client proteins demonstrated direct interactions with 14-3-3, exhibiting binding modes consistent with previously characterized 14-3-3/peptide complexes. This highlights the potential of our approach for identifying physiologically relevant phosphorylated binding sites within 14-3-3 client proteins.

## Conclusions and outlook

In this work, we developed and validated a deep learning approach for predicting putative protein–protein interaction sites between 14-3-3 proteins and phosphorylated client proteins. By leveraging different amino-acid sequence representations and neural network architectures, we demonstrated that our models outperform existing tools in terms of global performance, as captured by balanced accuracy. When combined within an ensemble model, our approach provided a robust predictive framework, enhancing the identification of novel binding sites for prospective applications by minimizing false positives compared to the state-of-the art.

Our model was applied to identify novel putative binding sites on biologically relevant 14-3-3 client proteins (FOXO3, Myc, BAD, Notch-4, CFTR and p53). The model was used to screen 296 potential binding sites and to select eight peptide sequences for follow-up assays. Experimental validation confirmed the predictive power of our model, with three out of eight newly predicted phosphopeptides exhibiting low-micromolar binding affinities to 14-3-3, two weak binders and two binders with marginal affinity. Structural characterization *via* X-ray crystallography further substantiated our findings, revealing binding modes consistent with known 14-3-3-client interactions. This includes an ‘open’ binding mode, where peptides bend out of the 14-3-3 pocket, an ‘extended’ binding mode, in which peptides occupy the entire 14-3-3 pocket, and peptides featuring a disordered C-terminus. The identification of such structurally representative 14-3-3 binding motifs, without having provided such structural information to our models, testifies to the strength of our deep learning approach. These findings were further corroborated by molecular dynamics simulations on longer peptide versions of the putative binding sites. Our study not only advances computational predictions for 14-3-3 interactions, but also underscores the importance of integrating deep learning with experimental validation. The results demonstrate that deep learning models can reliably predict potentially relevant binding sites for follow-up biological characterization, paving the way for more efficient exploration of the 14-3-3 interactome.

Several challenges and opportunities for future research remain. First, expanding the training dataset with additional experimentally validated binding and non-binding sequences will likely improve model generalizability. Incorporating sequence context beyond the immediate phosphosite region may further enhance predictive accuracy and facilitate the translation into biologically relevant insights. While our model effectively predicts linear phosphopeptide binding motifs, potentially ideal for disordered binding partners undergoing protein–protein interactions, future work could integrate structural data more comprehensively, potentially by incorporating protein tertiary and especially quaternary structure information. Combined, this might strongly aid addressing the challenge of refining interaction predictions for disordered regions and transient interactions.

Applying this predictive framework to other phospho-dependent interactions beyond 14-3-3 proteins could broaden its impact, aiding in the discovery of new regulatory mechanisms and therapeutic targets. Additionally, prospective validation of predicted binding sites in cellular models and *in vivo* systems will be necessary to fully establish the physiological relevance of our findings. Our approach contributes to a deeper understanding of peptide–14-3-3 interactions – supporting the rational design of modulators, and expanding the available hypotheses on 14-3-3 related cellular signaling. Furthermore, by making our model freely available on an online platform (https://14-3-3-bindsite.streamlit.app/), without requiring expert deep learning knowledge, we provide an accessible tool for researchers to explore 14-3-3 interactions in their own studies, fostering further discoveries in the field.

## Materials and Methods

### Data collection and curation

#### Publicly available data

We used a previously curated 14-3-3 binding site dataset,^26^ comprising 338 experimentally determined binding phosphosites,^73^ 93 experimentally determined non-binding phosphosites^74^ and 22 known binding sequences from the literature.^26^ Moreover, 262 likely non-binding phosphosites obtained randomly were added from proteins of which already two 14-3-3-binding sites were defined. In total the data contained 360 sequences labelled as binding and 355 labelled as non-binding. Sequences were centered around the phosphorylated AA and truncated or padded to 15 AAs, if necessary.

#### In-house test set

An in-house dataset of 92 phosphopeptides tested for binding to 14-3-3 was used for model evaluation. 58 of those phosphopeptides are annotated as binders (*K*_d_ < 200 μM) and 34 were annotated as non-binders (*K*_d_ > 200 μM). In cases with multiple affinity scores for different 14-3-3 isoforms, the strongest binding affinity was picked. Last, we centered the sequences around the phosphorylated residues to comply with the training set format, considered 15 AAs, and applied padding when necessary.

### Model training

#### Training and hyperparameter tuning

The dataset was split using 10-fold stratified cross-validation splitting. 10% was used as the test set and the remaining data was split into training and validation (67.5% and 22.5% of the total dataset, respectively). Test peptides with an edit distance on the AA sequence equal to or lower than four were removed to avoid data leakage or overestimation of model performance. We used a two-staged approach for hyperparameter tuning. First, a ‘broad’ hyperparameter space was tested (as recently suggested^75^), and, later, the top hyperparameter configurations (216 for GRU, 324 for MLP, and 1500 for CNN) were further fine-tuned (SI Table S6). Early stopping on *F*1 score was used starting from the fifth epoch, with a patience of five epochs. The model with the highest *F*1 score (eqn (5)) in 10-fold validation was selected. The final hyperparameters for each model are reported in SI Table S7.

#### Evaluation metrics

The capacity of the model to correctly classify positive (binding) and negative (non-binding) peptides was quantified *via* recall (Rc), precision (Pr) and specificity (Sp), computed as follows:^76^1
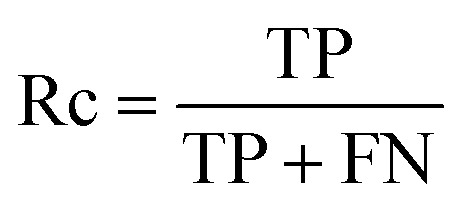
2
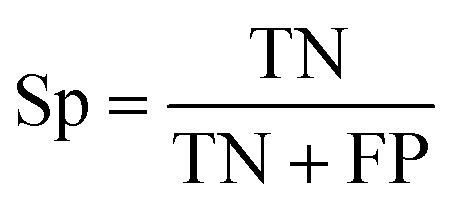
3
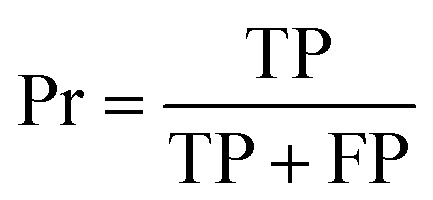
where true negatives (TN) and true positives (TP) represent the number of correctly identified non-binders and binders, respectively. Conversely, false negatives (FN) and false positives (FP) refer to the number of binders and non-binders that are misclassified. Recall (eqn (1)) indicates the proportion of actual binders that the model successfully identifies, specificity (eqn (2)) assesses the reliability of non-binding predictions, and precision (eqn (3)) measures the capability to minimize false positives.

Moreover, the models were assessed for their global prediction ability, *via* balanced accuracy (BA) and *F*1-score:4
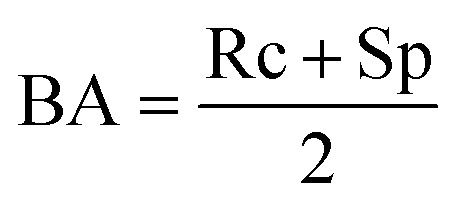
5
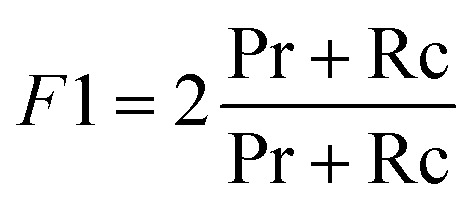


Balanced accuracy captures the model performance (correct predictions) normalized by the class imbalance, and *F*1 scores provide an overall evaluation of the model's performance in terms of minimizing false positives and negatives.

#### Peptide representation

The following settings were used for each peptide representation.

• One-hot-encoding. Each AA is assigned a unique vector with a single 1 corresponding to the respective index of that amino acid in the amino acid alphabet, with values of 0 in the remaining elements. Phosphorylated amino acids were encoded as a distinct token (and the corresponding sparse vector).

• Learnable embeddings. AAs are assigned a unique and randomly initialized vector. Phosphorylated AAs are assigned their own random vector. Vectors are then updated during model training.

• BLOSUM 62 representation was tested in two formats: (a) by treating phosphorylated AAs as their non-modified versions, and (b) by appending an additional numerical flag ([1,0] for phosphoserine and [0,1] for phosphothreonine). Preliminary results showed that the flagged version performed better in terms of *F*1 and balanced accuracy and hence it was used for this study.

• Peptide descriptors. For each AA, 18 descriptors were computed using the peptidy^32^ software. Descriptors were concatenated, obtaining a matrix of 15 × 18 descriptors per peptide (see SI Table S1).

### Prospective screening on selected proteins

#### Library preparation

The AA sequence for the selected proteins was obtained from UniProt^50^ (UniProt IDs: Tau = P10636-8; Myc = P01106; FOXO3 = O43524; Notch4 = Q99466; BAD = Q92934; CFTR = P13569; p53 = Q761V2). All serine and threonine residues were located and a window of 15 AAs was obtained (−7 and +7 around such AAs), leading to a total of 830 sequences. These sequences were further analyzed with PhosphoSitePlus,^51,77^ to predict whether they are phosphorylated *in vivo*. Only sequences phosphorylated were according to literature or PhosphoSitePlus were retained, resulting in a library of 296 peptides. All sequences were predicted with the ensemble model and ranked by scores (average predictions across the models). The top scoring predictions were manually inspected, and known binding sites identified and validated according to existing literature were excluded from the wet-lab validation (SI Table S4).

### Experimental validation

#### Peptide materials

Selected peptides were ordered from GenScript^78^ with a minimal purity of 85% with a N-terminal 6-aminohexanoic acid (Ahx) linker followed by the fluorescent dye 5-FAM. A C-terminal amidation served to mimic the lack of a free C-terminus in the amino-acid sequence when it is part of a larger protein. One of the top-scoring sequences was not tested due to failed synthesis by the commercial provider, and the next top-ranking sequence was picked instead. Acetylated peptides were ordered for crystallography with a minimal purity of 95%. Peptide sequences are reported in SI Table S8.

#### Protein expression and purification

The full-length (FL) human 14-3-3γ protein was expressed from a pPROEX plasmid after transformation to BL21(DE3) competent *E. coli* (Novagen). Cultures were incubated at 37 °C, 140 rpm until OD6_00_ ∼ 0.8 was reached. Protein expression was induced by isopropyl β-d-1-thiogalactopyranoside (IPTG; 0.4 mM) and cells were harvested by centrifugation (10 min, 4 °C, 16 000 × *g*) after overnight expression (18 °C, 140 rpm). Pellets were resuspended in wash buffer (50 mM Tris pH 8.0, 300 mM NaCl, 12.5 mM imidazole and 2 mM β-mercaptoethanol (βME)). After homogenizing the cells (40 bar, Emulsiflex-C3 homogenizer), the soluble fraction was collected by centrifugation (30 min, 4 °C, 40 000 × *g*) and loaded onto a Ni^2+^-affinity column pre-equilibrated with wash buffer. After a washing step (wash buffer + 20 mM imidazole), the bound protein was eluted with 200 mM imidazole followed by dialysis overnight at 4 °C (25 mM HEPES pH 8.0, 200 mM NaCl, 10 mM MgCl_2_, 0.5 mM tris(2-carboxyethyl)phosphine (TCEP)). The His_6_-tag of the Δ*C* variant (14-3-3σ truncated after S232) for crystallography was cleaved with TEV-protease during dialysis and subjected to an additional purification by size exclusion chromatography (SEC; Superdex 75; buffer 20 mM HEPES pH 7.5, 100 mM NaCl, 10 mM MgCl_2_, 0.5 mM TCEP). The pure protein was concentrated, aliquoted, flash-frozen in liquid N_2_, and stored at −80 °C. Purity and exact mass were determined (SI Fig. S6) using a high-resolution liquid chromatography coupled with mass spectrometry (LC/MS) system comprised of an I-Class Acquity UPLC (Waters) with a Polaris C18A reverse-phase column 2.0 × 100 mm (Agilent), coupled to a Xevo G2 Quadrupole Time of Flight mass spectrometer (Waters). A flow rate of 0.3 mL min^−1^ was used with a gradient of acetonitrile + 0.1% formic acid (FA) in water + 0.1% FA (acetonitrile 15–75%). Deconvolution of the *m*/*z* spectra was done using the MaxENT_I_ algorithm in the Masslynx v4.1 (SCN862) software.

#### Fluorescence anisotropy assay

To study the binding of the fluorescently labelled peptides to 14-3-3, Fluorescence Anisotropy (FA) assays were carried out.^23^ In the case of binding, tumbling of the peptide with the attached fluorophore will slow down and the emitted light will be polarized. This will lead to a higher anisotropy.^79^ For all experiments, 14-3-3γ was used as it was shown in multiple experiments to be the strongest binding variant suitable for experimental screening.^80^ The FAM-labeled peptides and the 14-3-3γ FL protein were diluted in buffer (10 mM HEPES pH 7.4, 150 mM NaCl, 0.1% Tween20, 1 mg mL^−1^ BSA).

Dilution series of 14-3-3γ proteins (starting at 500 μM) were made to 10 nM of the FAM-labeled peptides in black, round-bottom 384-microwell (Corning) in a final sample volume of 10 μL. Fluorescence anisotropy values were measured using a Tecan Spark Control at room temperature filter set lex: 485 ± 20 nm, lem: 535 ± 25 nm, mirror: Dichroic 510, flashes: 30, integration time: 40 μs, settle time: 1 μs; gain: optimized per peptide, and *Z*-position: calculated from well. Wells containing only FAM-peptide were used to set as *G*-factor. The *K*_D_ values were obtained from fitting the data using Origin 2020 with a Sigmoid Hill1 function (using the Hill equation). Data shown is the average and standard deviation of triplicates. For dose–response curves on a linear scale seep SI Fig. S2.

### Co-crystallization

The 14-3-3σΔC protein and the acetylated client peptides were dissolved in complexation buffer (25 mM HEPES pH = 7.5, 2 mM MgCl_2_ and 100 μM TCEP) and mixed in a 1 : 2 or 1 : 4 molecular stoichiometry (protein : peptide) with a final protein concentration of 12 mg mL^−1^. The complex was set-up for sitting-drop crystallization at 4 °C, in a custom crystallization liquor (0.05 M HEPES (pH 7.1, 7.3, 7.5, 7.7), 0.19 M CaCl_2_, 24–29% PEG400, and 5% (v/v) glycerol). Crystals grew within 10–14 days at 4 °C. Crystals were fished and flash-cooled in liquid nitrogen. X-ray diffraction (XRD) data were collected at the European Synchrotron Radiation Facility (ESRF Grenoble, France, beamline ID23-2). Data was processed using CCP4i2 suite (version 8.0.019). After indexing and integrating the data, scaling was done using AIMLESS. The data was phased with MolRep, using PDB 3IQU as template. Model rebuilding and refinement was performed using REFMAC5. The PDB REDO server (https://pdb-redo.edu) was used to complete the model building and refinement. The images were created using the PyMOL Molecular Graphics System (Schrödinger LLC, version 4.6.0). See SI Table S9 for data collection and refinement statistics. The structures were deposited in the protein data bank (PDB) with IDs: **9QNG** (FOXO pS413), **9QNH** (Myc pS294), **9QNI** (NOTCH4 pS1847), **9QNJ** (Tau pS198), **9QNK** (Tau pT245), **9QNL** (BAD pS118).

### Molecular dynamics simulations

To investigate how our experimental results could be extended beyond the experimentally determined peptides, we selected three sequences: FOXO3 pS413 (1, *K*_d_ = 1.6 ± 0.1 μM), TAU pT245 (2, *K*_d_ = 8.5 ± 0.2 μM), and BAD pS118 (7, *K*_d_ > 100 μM). For each of these sequences, we obtained an ‘extended’ sequence from the corresponding full-protein sequence from UniProt,^50^ by elongating the tested sequences with 20 AAs in both N- and C-terminal directions (55 AAs in total). Our goal was to assess whether the additional flanking residues could alter the binding properties of the peptide within the 14-3-3 binding pocket and hence infer the plausibility of the predicted binding sites. To this end, we performed molecular dynamics (MD) to compare the stability of both sequence versions, ultimately to assess how additional flanking residues influence stability and to gain insights into these PPIs. For each peptide (initial sequence), molecular dynamics (MD) simulations were performed using GROMACS 2023 ^81^ with three independent replicates. The simulations were divided into three stages: energy minimization, equilibration, and production. Energy minimization was performed using the steepest descent algorithm until a convergence criterion of 1000 kJ mol^−1^ nm^−1^ was reached. The equilibration phase was conducted under position-restrained dynamics in the NVT and NPT ensembles, using the V-rescale thermostat to maintain a temperature of 303.15 K and the Parrinello-Rahman barostat to regulate pressure at 1 atm. The production phase involved MD simulations for 300 ns with a 2 fs integration time step.

Following, the initial peptide structures were extended to a sequence of 55 AAs using PyMOL.^82^ MD simulations were performed following a five-step protocol to ensure proper system relaxation and equilibration. The first step involved an energy minimization using steepest descent, applying positional restraints on the backbone (force constant = 400 kJ mol^−1^ nm^−2^) and side chains (force constant = 40 kJ mol^−1^ nm^−2^). The peptide was frozen along all spatial dimensions during this phase. In the second step, a 5 ns MD simulation was carried out under NVT conditions, with positional restraints on the backbone and side chains. A time step of 1 fs was used, and the system was maintained at 303.15 K using the V-rescale thermostat. The peptide remained frozen along all spatial dimensions. Following this, a second round of energy minimization was performed using the same parameters as in the first phase to allow for further relaxation of the solvent environment around the peptide. The fourth phase involved a 5 ns MD simulation under NPT conditions to equilibrate the system. Positional restraints were again applied to the peptide backbone and side chains. Pressure was controlled isotropically at 1 bar using the Parrinello-Rahman barostat, and temperature was held at 303.15 K using the V-rescale thermostat. Finally, in the fifth phase, a 300 ns production MD simulation was carried out with a 2 fs time step, during which positional restraints were removed, allowing the peptide to move freely. Temperature (303.15 K) and pressure (1 bar) were controlled using the V-rescale thermostat and Parrinello-Rahman barostat,^83^ respectively. For the analysis of the root mean square fluctuation (RMSF) of the peptides, the first 15 ns of the production phase were excluded from the calculation to allow for system equilibration. The RMSF values were then computed over the remaining trajectory, considering the fluctuations across all three replicates.

## Author contributions

Conceptualization: all authors; methodology (computational): RÖ, LvW, EC, FG; methodology (experimental): LvW, MP, CO, LB; software: LvW, RÖ; validation (computational): LvW, RÖ, EC; validation (experimental): LvW, MP; formal analysis: all authors; visualization: FG, LvW, MP, RÖ, and EC; resources and funding acquisition: FG and LB; supervision: FG, LB, CO; writing – original draft: LvW, FG, RÖ, with contributions from MP and EC; writing – review & editing: all authors.

## Conflicts of interest

CO and LB are co-founders of Ambagon Therapeutics. The other authors declare no conflict of interest.

## Supplementary Material

DD-004-D5DD00132C-s001

## Data Availability

The Python code and the data to replicate and extend our study are available on GitHub at the following URL: https://github.com/molML/14-3-3-bindsite. To further apply our approach prospectively, the software can be used *via* a freely accessible webpage at the following URL: https://14-3-3-bindsite.streamlit.app/. The code and data associated with this work have been deposited in Zenodo at the time of publication; they can be accessed *via* the following DOI: https://doi.org/10.5281/zenodo.16420029. Supplementary tables (Tables S1–S9) and figures (Fig. S1–S6) are available. See DOI: https://doi.org/10.1039/d5dd00132c.
