# Supplementary material for: Identifying 14-3-3 interactome binding sites with deep learning
Source: Digit Discov. 2025 Aug 8;4(9):2602–14. doi: 10.1039/d5dd00132c (PMC12360161; doi:10.1039/d5dd00132c)
Supplement: DD-004-D5DD00132C-s001 [file DD-004-D5DD00132C-s001.pdf]

Supplementary Information (SI) for Digital Discovery.
This journal is © The Royal Society of Chemistry 2025

Supporting Information

Identifying 14-3-3 interactome binding sites with deep learning

Laura van Weesep, Rıza Özçelik, Marloes Pennings, Emanuele Criscuolo, Christian Ottmann, Luc Brunsveld and Francesca Grisoni

Supplementary Tables

Supplementary Table S1. List of psycho-chemical properties computed per amino-acid, using peptidy.[1]

1. Aliphatic Index	7. No. Carbon Atoms	13. Molecular Weight
2. Aromaticity	8. No. Hydrogen Atoms	14. No. Hydrogen Bond Donors
3. Charge	9. No. Nitrogen Atoms	15. No. Hydrogen Bond Acceptors
4. Charge Density	10. No. Oxygen Atoms	16. Topological Polar Surface Area
5. Hydrophobic AA Ratio	11. No. Sulfur Atoms	17. Energy Based on LogP
6. Isoelectric Point	12. No. Phosphor Atoms	18. Average No. Rotatable Bonds

Supplementary Table S2. Machine learning performance evaluation on the test set. Classification metrics are reported as the mean and standard deviation across 10 training and test splits after hyperparameter tuning.

Architecture	Representation	BA (%)	Pr (%)	Recall (%)	Specificity (%)
**MLP**	Learnable	77±4	**76±6**	80±10	74±9
**CNN**	BLOSUM	73±5	71±5	80±7	66±8
**GRU**	BLOSUM	**78±6**	77±6	79±10	**77±8**
**Ensemble**	**//**	77±5	75±6	**82±9**	72±8

Supplementary Table S3. Intrinsic disorder ratio, as prediced by AlphaFold (and retrieved on MobiDB; https://mobidb.org, accessed February 2025) or experimentally determined (retrieved via DistProt; https://disprot.org/, accessed February 2025). UniProtID was used for retrieval.

Protein	UniProtID	MobiDB (AlphaFold)	DistProt (experimental)
p53	P04637	44.30%	37.7%
CFTR	P13569	23.20%	12.50%
Notch4	Q99466	25.50%	n.a.
Myc	P01106	69.60%	57.86%
FOXO3	O43524	85.30%	n.a.
BAD	Q92934	85.70%	n.a.
Tau	P10636-8	95.10%	n.a.

Supplementary Table S4. Known binding sites that were correctly predicted by our model.

Protein	Phospohosite	Model Prediction	Reference
Tau	214	0.98±0.03	[2,3]
	324	0.66±0.14	[3]
	356	0.70±0.21	[4]
BAD	74	0.77±0.18	[5]
	75	0.96±0.05	[6] (mice)
	99	1.0±0.0	[7,8]
FOXO3	32	0.88±0.14	[9]
	253	0.99±0.01	[10]
Notch-4	1865	0.89±0.10	[11]
CFTR	737	0.70±0.19	[12]
	768	0.88±0.09	[12]
Myc	358	0.74±0.15	Truncated version tested in house
p53	366	0.85±0.14	[13]

Supplementary Table S5. Ranking of the ordered peptides according to our model and the model available on the 14-3-3 Site Finder. For the other models the output from 14-3-3 site finder was used. Correlation (*r*) is computed between our ranking and those of the other methods. *Rank out the 296 phosphorylated peptides based on the 7 proteins tested. *Out of 418 top 50% entries. For all models, the Uniprot ID input used to retrieve the full protein sequences is: P10636-8,P01106,O43524,Q99466,Q92934,P13569,P04637.
*** Correlation with the ranking based on Kd values.

**Protein**	**Phospho -site**	**This work*** [rank, ID]	**Total score 14-3-3 site finder****[14]	**14-3-3 Pred****[15]	**Adapted 14-3-3 Pred score****[14]	**K_d (µM)**
FOXO 3	413	3 (**1**)	10	16	2	1.6 ± 0.1 (1)
Tau	245	6 (**2**)	15	55	10	8.6 ± 0.8 (2)
Notch 4	1847	10 (**3**)	44	23	24	70 ± 1 (4)
Tau	198	11 (**4**)	36	184	104	71 ± 11 (5)
CFTR	422	20 (**5**)	76	21	40	– (8)
BAD	134	21 (**6**)	14	80	15	15.9 ± 1.9 (3)
BAD	118	23 (**7**)	37	86	20	> 100 (6)
Myc	294	24 (**8**)	84	98	25	> 100 (7)
Corr.		1.00	0.61	0.17	0.05	0.74***

Supplementary Table S6. Hyperparameter space analyzed for the second round of hyper-parameter tuning each model (n.a. = not applicable). The search strategy (random or exhaustive) along with the number of tested hyperparameter combinations was reported.

Hyperparameter	MLP	CNN	GRU
Activation	ReLu	ReLu	ReLu
No. (dense) layers	1, 2, 3	1, 2, 3, 4	1, 2
No. of neurons per (dense) layer	8, 20, 64, 128	16, 64, 256	32, 64, 128, 256
Learning rate	1E-2, 1E-3, 5E-3	1E-2, 1E-3, 5E-3	1E-2, 1E-3, 5E-3
Batch size	32, 64, 128	32, 64, 128	32, 64, 256
Dropout	0.0, 0.1, 0.25	0.0, 0.1, 0.25	0.0, 0.1, 0.25
Epochs	200	200	200
Loss	BCE	BCE	BCE
Embedding size	32, 64	32, 64	32, 64
No. of 1D layers	n.a.	Other: 2, 3, 4	n.a.
Kernel size of the 1D layers	n.a.	5, 7, 9	n.a.
Number of filters	n.a.	3, 5, 7	n.a.
Search strategy	Exhaustive (except embedding)	Random	Exhaustive (except embedding)
No. HP combinations	324	1500	216

Supplementary Table S7. Selected model hyperparameters (n.a*.* = not applicable).

**Hyperparameter**	**MLP**	**CNN**	**GRU**
No. dense layers	2	4	1
No. neurons per dense layer	20	256	1
Learning rate	0.001	0.01	0.001
Batch size	128	64	128
Dropout	0.0	0.25	0.25
Epochs	18	37	25
Loss	BCE	BCE	BCE
Batch normalization	False	True	False
Embedding size	32	*n.a.*	n.a.
No. 1D conv. layers	n.a.	3	n.a.
Kernel size of the 1D layers	n.a.	5	n.a.
Number of filters	n.a.	5	n.a.

Supplementary Table S8. Ordered peptide sequences.

FOXO pS413	5-FAM-Ahx-GLMQRSS**pS**FPYTTKG-CONH2
Tau pT245	5-FAM-Ahx-SAKSRLQ**pT**APVPMPD-CONH2
NOTCH4 pS1847	5-FAM-Ahx-FPRARTV**pS**VSVPPHG-CONH2
Tau pS198	5-FAM-Ahx-SGDRSGY**pS**SPGSPGT-CONH2
Myc pS294	5-FAM-Ahx-APGKRSE**pS**GSPSAGG-CONH2
BAD pS134	5-FAM-Ahx-KGLPRPK**pS**AGTATQM-CONH2
BAD pS118	5-FAM-Ahx-GRELRRM**pS**DEFVDSF-CONH2
CFTR pS422	5-FAM-Ahx-NNNNRKT**pS**NGDDSLF-CONH2
Tau pT111	5-FAM-Ahx-EEAGIGD**pT**PSLEDEA-CONH2
Myc pT262	5-FAM-Ahx-LHEETPP**pT**TSSDSEE-CONH2
CFTR T1019	5-FAM-Ahx-QPYIFVA**pT**VPVIVAF-CONH2 (*Synthesis failed*)

Supplementary Table S9. XRD data collection and refinement statistics for 14-33σ/peptide structures.

**PDB**	**9QNG**	**9QNK**	**9QNI**
Protein	14-3-3sDC	14-3-3sDC	14-3-3sDC
Peptide	FOXO3 pS413	Tau pT245	NOTCH4 pS1847
Beam	ESRF ID23-2	ESRF ID23-2	ESRF ID23-2
DOI	10.2210/pdb9qng/pdb	10.2210/pdb9qnk/pdb	10.2210/pdb9qni/pdb
Data collection			
Wavelength (Å)	0.873128	0.873128	0.873128
Space group	C 2 2 21	C 2 2 21	C 2 2 21
Cell dimensions<br>a, b, c (Å)<br>α, β, γ (°)	 82.1, 112.0, 63.1<br>90, 90, 90	 82.9, 113.0, 63.2<br>90, 90, 90	 83.1, 113.0, 63.3<br>90, 90, 90
Resolution (Å)	63.07 – 1.35<br>(1.37 – 1.35)	45.92 – 1.6<br>(1.63 – 1.6)	66.93 – 1.8<br>(1.84 - 1.8)
I / σ(I)	14.3 (1.0)	14.6 (2.4)	16.5 (2.8)
Completeness (%)	100.0 (100.0)	86.5 (100.0)	82.3 (100)
Redundancy	12.9 (12.7)	12.3 (12.0)	9.0 (9.6)
$CC_{1/2}$	0.999 (0.394)	0.999 (0.777)	0.999 (0.805)
Refinement			
No. reflections	63042	34167	23160
R_{work}/R_{free}	0.161/0.189	0.201/0.224	0.185/0.220
No. atoms<br>Protein<br>Ligand/ion<br>Water	 1992<br>6<br>252	 1959<br>4<br>184	 1913<br>5<br>122
B-factors<br>Protein<br>Ligand/ion<br>Water	 23.36<br>34.04<br>35.47	 22.54<br>29.35<br>34.68	 34.16<br>44.49<br>35.83
R.m.s. deviations<br>Bond lengths (Å)<br>Bond angles (°)	 0.012<br>1.07	 0.016<br>1.37	 0.011<br>1.19
Ramachandran<br>favored (%)<br>outliers (%)	 98.74<br>0.00	 98.30<br>0.00	 97.85<br>0.43

**PDB**	**9QNJ**	**9QNL**	**9QNH**
Protein	14-3-3sDC	14-3-3sDC	14-3-3sDC
Peptide	Tau pS198	BAD pS118	Myc pS294
Beam	ESRF ID23-2	ESRF ID23-2	ESRF ID23-2
DOI	10.2210/pdb9qnj/pdb	10.2210/pdb9qnl/pdb	10.2210/pdb9qnh/pdb
Data collection			
Wavelength (Å)	0.873128	0.873128	0.873128
Space group	C 2 2 21	C 2 2 21	C 2 2 21
Cell dimensions<br>a, b, c (Å)<br>α, β, γ (°)	 82.6, 112.4, 63.0<br>90, 90, 90	 82.6, 112.1, 63.0<br>90, 90, 90	 82.9, 112.7, 63.0<br>90, 90, 90
Resolution (Å)	66.57 – 1.3 (1.32 – 1.3)	45.72 – 1.3 (1.33 – 1.3)	66.76 – 1.3 (1.33 – 1.3)
$I / \sigma(I)$	24.7 (2.7)	19.4 (1.5)	31.2 (5.4)
Completeness (%)	100 (100)	89.1 (97.9)	88.3 (98.1)
Redundancy	13.0 (12.8)	13.0 (12.5)	13.5 (13.1)
$CC_{1/2}$	1.000 (0.848)	1.000 (0.589)	1.000 (0.948)
Refinement			
No. reflections	72103	62166	63470
R_{work}/R_{free}	0.163/0.183	0.170/0.202	0.189/0.209
No. atoms<br>Protein<br>Ligand/ion<br>Water	 1996<br>8<br>262	 2007<br>6<br>238	 1991<br>8<br>280
B-factors<br>Protein<br>Ligand/ion<br>Water	 20.48<br>48.06<br>34.19	 25.25<br>38.83<br>35.68	 19.23<br>31.73<br>32.02
R.m.s. deviations<br>Bond lengths (Å)<br>Bond angles (°)	 0.018<br>1.43	 0.009<br>1.03	 0.009<br>0.99
Ramachandran<br>favored (%)<br>outliers (%)	 98.29<br>0.00	 98.32<br>0.00	 98.72<br>0.00

Supplementary Figures

Type	Training set	In-house
All	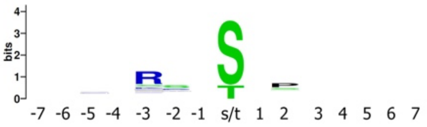	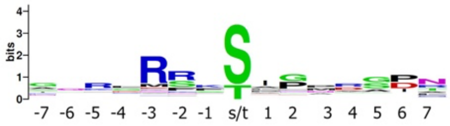
Binders	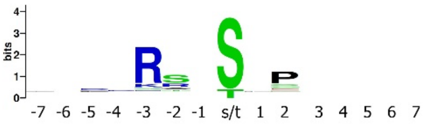	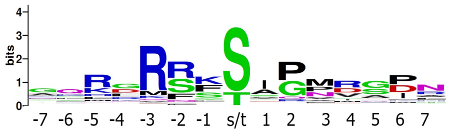
Non-binders	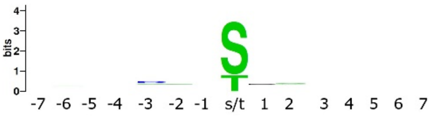	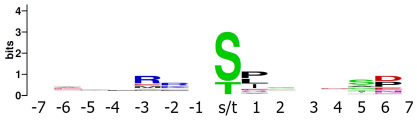

Supplementary Figure S1. LogoPlots of the sequences in literature dataset used to train and validate the models, divided by their binding label.

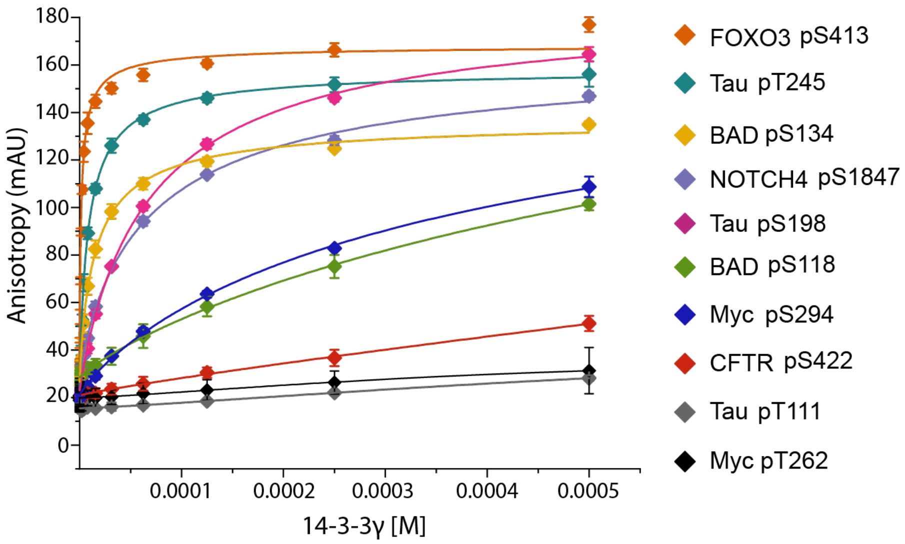

Supplementary Figure S2. Experimental validation. Eight putative binding sites (**1**-**8**) and two negative controls (**9**-**10**) were selected for experimental validation via fluorescence anisotropy (FA) assays. Dose-response curves are reported for each sequence, labeled as protein and phosphosite (pS = phosphoserine, pT = phosphothreonine), across three independent repeats. Plotted on linear scale, showing similar data as in Figure 2a (plotted on logarithmic scale).

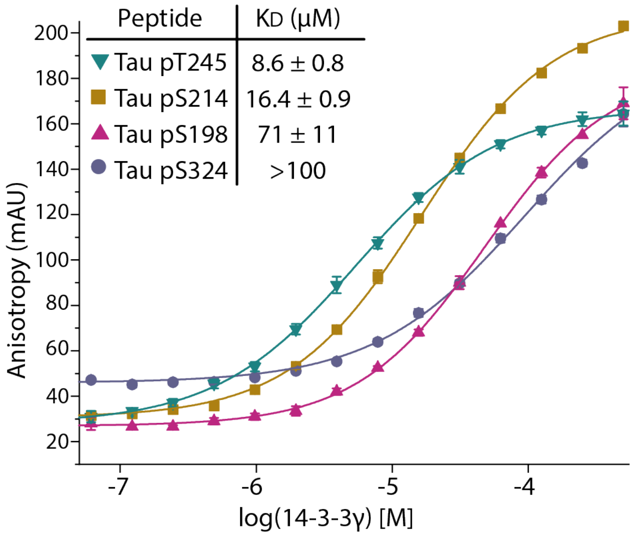

Supplementary Figure S3. Titration of 14-3-3y to fluorescently labeled peptides Tau pS198 and pT245, and two known Tau binding sites, Tau pS214 and pS324 (10 nM). Data and K_D values are shown as mean ± SD (n=3).

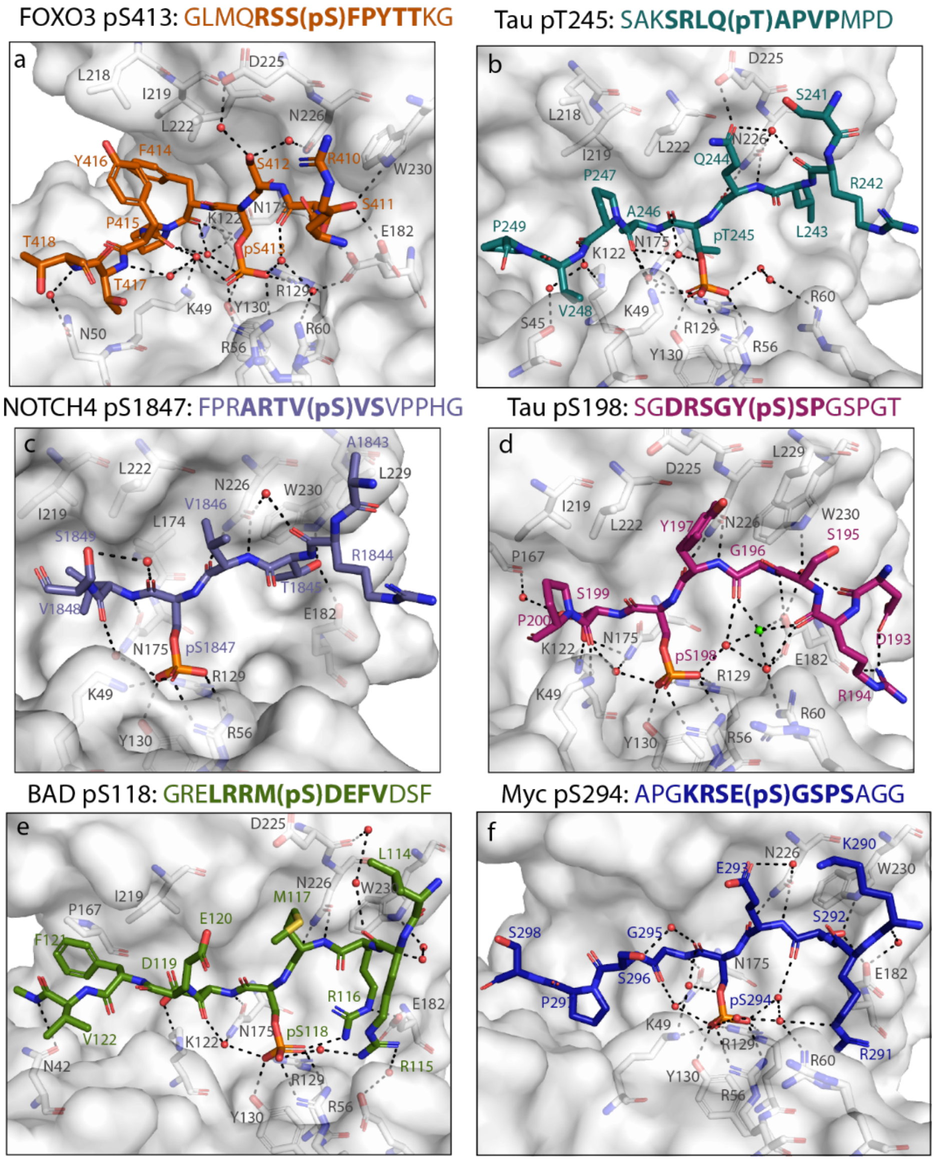

Supplementary Figure S4. Interactions of predicted peptide sequences (**a**) FOXO3 pS413 (orange), (**b**) Tau pT245 (cyan), (**c**) NOTCH pS1847 (purple), (**d**) Tau pS198 (pink), (**e**) BAD pS118 (green), (**f**) Myc pS294 (blue)) with 14-3-3sigma (white surface) (relevant side chains and waters are displayed as stick and red dots, respectively, polar contacts are shown as black dashed lines).

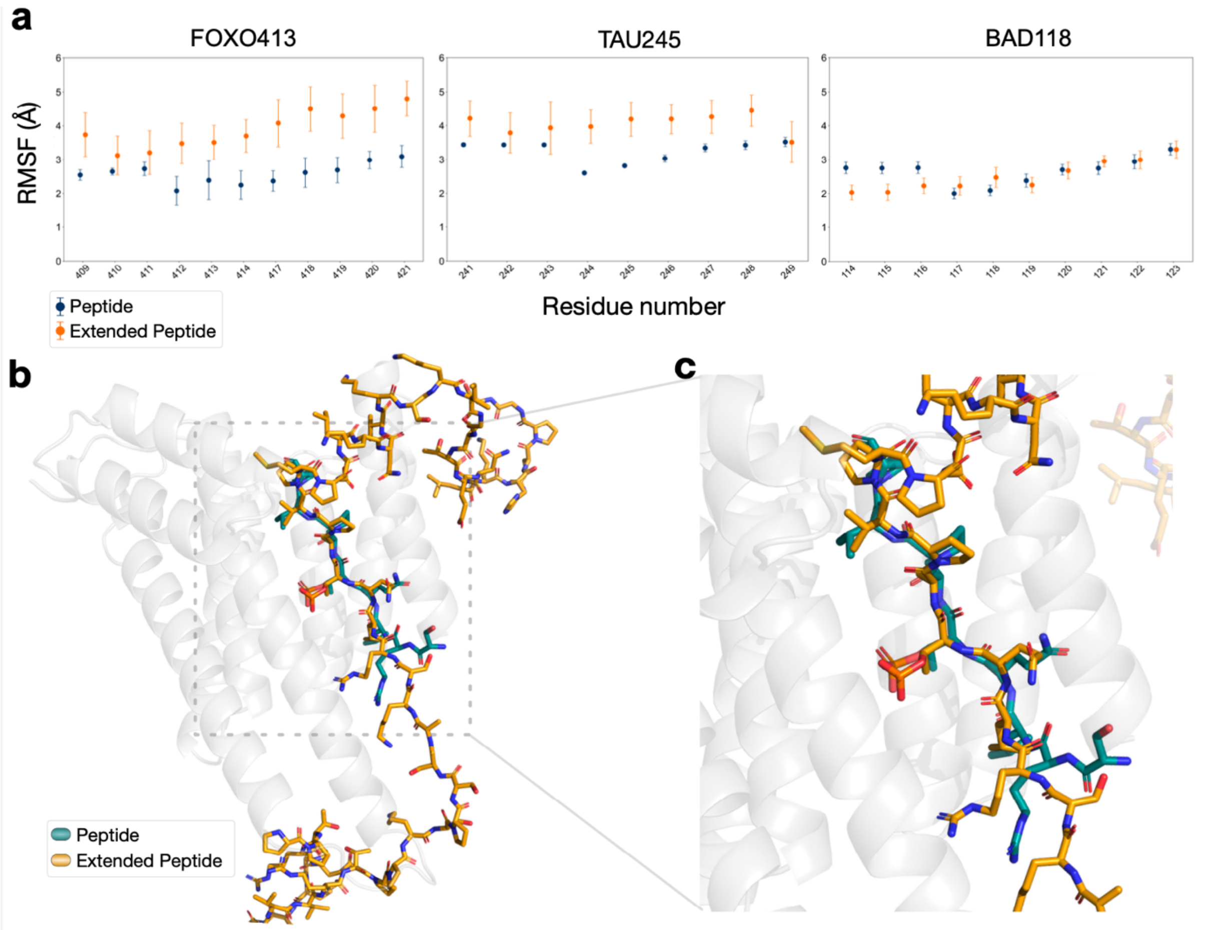

Supplementary Figure S5. Structural insights into binding to 14-3-3. Molecular dynamics analysis to determine the stability of putative binding sites, by comparing three tested peptides (FOXO3-413, TAU-245 and BAD-118) with an extended version along the respective protein sequence (55 AAs). (**a**) Root mean squared fluctuation (RMSF [Å] – the lower, the more stable) values obtained for the selected peptides and the extended AA sequence. RMSF is reported per residue. (**b**) Structural overlay of the TAU245 peptide and its extended form into the 14-3-3 binding site after molecular dynamics simulation. (**c**) The binding poses of TAU245 at the interface with 14-3-3 are conserved among the 15 AAs and the 55 AAs versions throughout the MD simulations.

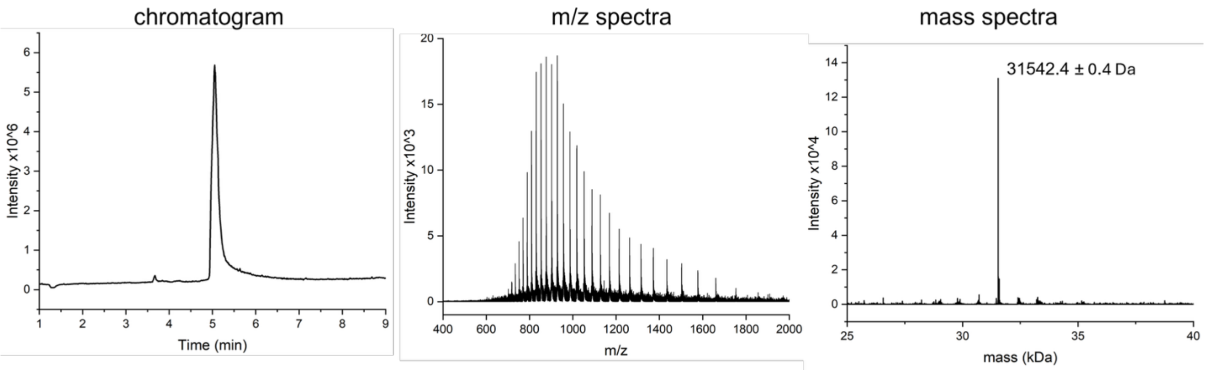

Supplementary Figure S6. Protein characterization by high-resolution liquid chromatography coupled with mass spectrometry (LC/MS) system. Chromatogram (left), deconvoluted m/z spectra (middle), and mass spectra (right) of purified 14-3-3 protein used in this study.

Reference

(1) Özçelik, R.; van Weesep, L.; de Ruiter, S.; Grisoni, F. Peptidy: A Light-Weight Python Library for Peptide Representation in Machine Learning. *ChemRxiv* **2024**. https://doi.org/10.26434/chemrxiv-2024-bm3lv.

(2) Sadik, G.; Tanaka, T.; Kato, K.; Yamamori, H.; Nessa, B. N.; Morihara, T.; Takeda, M. Phosphorylation of Tau at Ser214 Mediates Its Interaction with 14-3-3 Protein: Implications for the Mechanism of Tau Aggregation. *J. Neurochem.* **2009**, *108* (1), 33–43. https://doi.org/10.1111/j.1471-4159.2008.05716.x.

(3) Joo, Y.; Schumacher, B.; Landrieu, I.; Bartel, M.; Smet-Nocca, C.; Jang, A.; Choi, H. S.; Jeon, N. L.; Chang, K.-A.; Kim, H.-S.; Ottmann, C.; Suh, Y.-H. Involvement of 14-3-3 in Tubulin Instability and Impaired Axon Development Is Mediated by Tau. *FASEB J. Off. Publ. Fed. Am. Soc. Exp. Biol.* **2015**, *29* (10), 4133–4144. https://doi.org/10.1096/fj.14-265009.

(4) Sluchanko, N. N.; Seit-Nebi, A. S.; Gusev, N. B. Phosphorylation of More than One Site Is Required for Tight Interaction of Human Tau Protein with 14-3-3ζ. *FEBS Lett.* **2009**, *583* (17), 2739–2742. https://doi.org/10.1016/j.febslet.2009.07.043.

(5) Sluchanko, N. N.; Tugaeva, K. V.; Gushchin, I.; Remeeva, A.; Kovalev, K.; Cooley, R. B. Crystal Structure of Human 14-3-3ζ Complexed with the Noncanonical Phosphopeptide from Proapoptotic BAD. *Biochem. Biophys. Res. Commun.* **2021**, *583*, 100–105. https://doi.org/10.1016/j.bbrc.2021.10.053.

(6) Zha, J.; Harada, H.; Yang, E.; Jockel, J.; Korsmeyer, S. J. Serine Phosphorylation of Death Agonist BAD in Response to Survival Factor Results in Binding to 14-3-3 Not BCL-XL. *Cell* **1996**, *87* (4), 619–628.

(7) Datta, S. R.; Katsov, A.; Hu, L.; Petros, A.; Fesik, S. W.; Yaffe, M. B.; Greenberg, M. E. 14-3-3 Proteins and Survival Kinases Cooperate to Inactivate BAD by BH3 Domain Phosphorylation. *Mol. Cell* **2000**, *6* (1), 41–51. https://doi.org/10.1016/S1097-2765(05)00012-2.

(8) Masters, S. C.; Yang, H.; Datta, S. R.; Greenberg, M. E.; Fu, H. 14-3-3 Inhibits Bad-Induced Cell Death through Interaction with Serine-136. *Mol. Pharmacol.* **2001**, *60* (6), 1325–1331. https://doi.org/10.1124/mol.60.6.1325.

(9) Singh, A.; Ye, M.; Bucur, O.; Zhu, S.; Tanya Santos, M.; Rabinovitz, I.; Wei, W.; Gao, D.; Hahn, W. C.; Khosravi-Far, R. Protein Phosphatase 2A Reactivates FOXO3a through a Dynamic Interplay with 14-3-3 and AKT. *Mol. Biol. Cell* **2010**, *21* (6), 1140–1152.

(10) Mathivanan, S.; Lakshman, P. K. C.; Singh, M.; Giridharan, S.; Sathish, K.; Hurakadli, M. A.; Bharatham, K.; Kamariah, N. Structure of a 14-3-3ε:FOXO3apS253 Phosphopeptide Complex Reveals 14-3-3 Isoform-Specific Binding of Forkhead Box Class O Transcription Factor (FOXO) Phosphoproteins. *ACS Omega* **2022**, *7* (28), 24344–24352. https://doi.org/10.1021/acsomega.2c01700.

(11) Ramakrishnan, G.; Davaakhuu, G.; Chung, W. C.; Zhu, H.; Rana, A.; Filipovic, A.; Green, A. R.; Atfi, A.; Pannuti, A.; Miele, L.; Tzivion, G. AKT and 14-3-3 Regulate Notch4 Nuclear Localization. *Sci. Rep.* **2015**, *5* (1), 8782. https://doi.org/10.1038/srep08782.

(12) Stevers, L.; Lam, C. V.; Leysen, S.; Meijer, F.; Scheppingen, D.; de Vries, R.; Carlile, G.; Milroy, L.-G.; Thomas, D.; Brunsveld, L.; Ottmann, C. Characterization and Small-Molecule Stabilization of the Multisite Tandem Binding between 14-3-3 and the R Domain of CFTR. *Proc. Natl. Acad. Sci. U. S. A.* **2016**, *113*. https://doi.org/10.1073/pnas.1516631113.

(13) Rajagopalan, S.; Sade, R. S.; Townsley, F. M.; Fersht, A. R. Mechanistic Differences in the Transcriptional Activation of P53 by 14-3-3 Isoforms. *Nucleic Acids Res.* **2009**, *38* (3), 893–906. https://doi.org/10.1093/nar/gkp1041.

(14) Egbert, C. M.; Warr, L. R.; Pennington, K. L.; Thornton, M. M.; Vaughan, A. J.; Ashworth, S. W.; Heaton, M. J.; English, N.; Torres, M. P.; Andersen, J. L. The Integration of Proteome-Wide PTM Data with Protein Structural and Sequence Features Identifies Phosphorylations That Mediate 14-3-3 Interactions. *J. Mol. Biol.* **2023**, *435* (2), 167890. https://doi.org/10.1016/j.jmb.2022.167890.

(15) Madeira, F.; Tinti, M.; Murugesan, G.; Berrett, E.; Stafford, M.; Toth, R.; Cole, C.; MacKintosh, C.; Barton, G. J. 14-3-3-Pred: Improved Methods to Predict 14-3-3-Binding Phosphopeptides. *Bioinformatics* **2015**, *31* (14), 2276–2283. https://doi.org/10.1093/bioinformatics/btv133.
